# Ureteropelvic Junction Obstruction and Parathyroid Adenoma: Coincidence or Link?

**DOI:** 10.1155/2017/9852912

**Published:** 2017-10-17

**Authors:** Salah Termos, Majd AlKabbani, Tim Ulinski, Sami Sanjad, Henri Kotobi, Francois Chalard, Bilal Aoun

**Affiliations:** ^1^Hepatobiliary and Transplant Unit, Department of Surgery, Al-Amiri Hospital, Kuwait City, Kuwait; ^2^Pediatric Nephrology, Armand Trousseau Hospital, APHP, Paris, France; ^3^Division of Pediatric Nephrology, Department of Pediatrics, American University of Beirut, Beirut, Lebanon

## Abstract

Congenital ureteropelvic junction obstruction (UPJO) is the most common cause of upper urinary tract obstruction in children. It is generally diagnosed in the routine work-up during antenatal period and is characterized by spontaneous recovery. It can be associated with urolithiasis; hence further investigation should be carried out. We report the case of a 15-year-old boy, who is known to have right UPJO, presented with right renal colic and discovered to have bilateral kidney stones. Further studies showed primary hyperparathyroidism and genetic analysis revealed a CDC73 mutation (initially HRPT2). We believe that association of UPJO and PHPT is a rare coincidence that can be linked. Careful work-up of children with UPJO and urolithiasis is recommended to exclude an underlying metabolic disease. Surgical correction can be evitable as treatment of the primary cause can lead to complete dissolution of kidney stones and improvement of the medical condition.

## 1. Introduction

Ureteropelvic junction obstruction (UPJO) has a reported incidence of 1 in 500 live births [1], more commonly in males than females and more frequently found on the left side. It can be congenital or acquired, but congenital cases are more common. It is considered the most common cause of antenatally detected hydronephrosis [2, 3].

Management of UPJO depends on symptoms and split renal function and it includes conservative management with observation and follow-up or surgical intervention. UPJO can lead to urolithiasis due to obstruction and urinary stasis; however, metabolic causes of urolithiasis should be investigated and ruled out [4–6]. We describe an unusual case of UPJO associated with PHTP and kidney stones.

## 2. Case Presentation

In our manuscript, we report the case of a 15-year-old boy with a longstanding history of unilateral ureteropelvic junction obstruction who was presented for right flank pain of three-month duration. The patient had been followed up for his right UPJO since birth, as he was diagnosed prenatally to have hydronephrosis. An early ultrasound imaging of the kidney was done at the age of three months and revealed a right renal pelvis dilatation of 15 mm (anteroposterior diameter) with normal kidney parenchyma. Later at the age of three years, a follow-up ultrasound noted an increased dilatation of the right pelvis up to 20 mm. Further studies were carried out; a MAG-3 scintigraphy was performed and showed a good contrast evacuation (10% residual radioactivity, 20 minutes after furosemide injection) and symmetric kidney function (45% for the right kidney and 55% for the left). Furthermore, the child was followed regularly with renal ultrasound that revealed a stationary course of pelvic dilation within 15–20 mm without any clinical manifestation.

**Figure 1:**
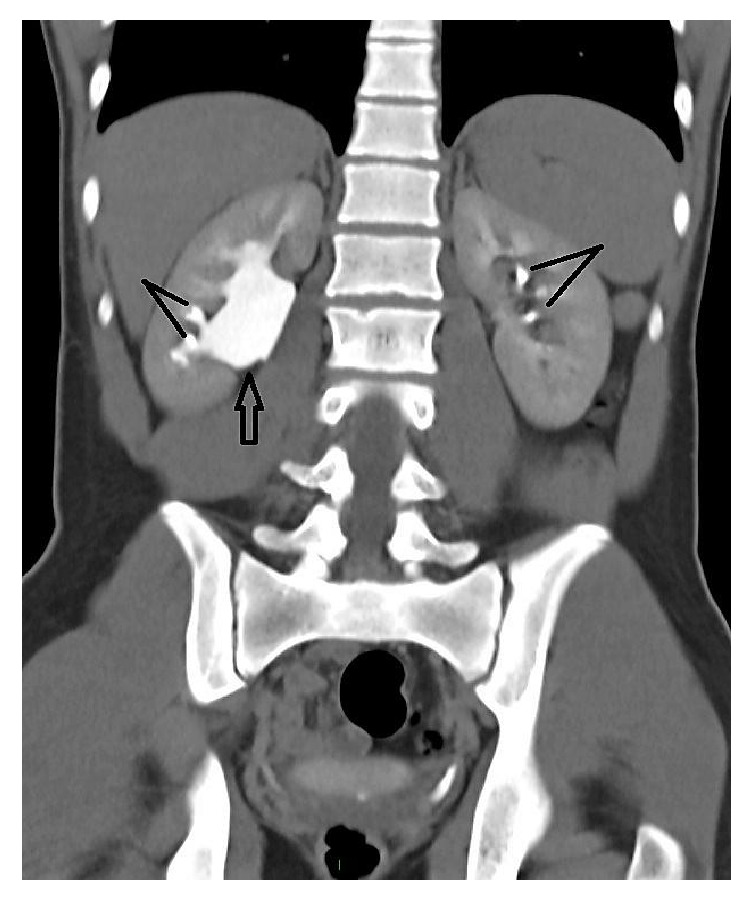
CT-scan of the abdomen and pelvis demonstrating moderately dilated right renal pelvis of 19 mm (headed arrow) and bilateral renal stones (dashes).

At the age of 15 years, the patient presented to our institution for right flank pain, without urinary symptoms. Renal ultrasound showed bilateral kidney stones (8 to 9 mm). A CT-scan of the abdomen showed a moderately dilated right pelvis of 19 mm containing three stones, in addition to two stones in a nondilated left renal pelvis (Figure 1). There was also a lytic lesion in the right iliac bone.

**Figure 2:**
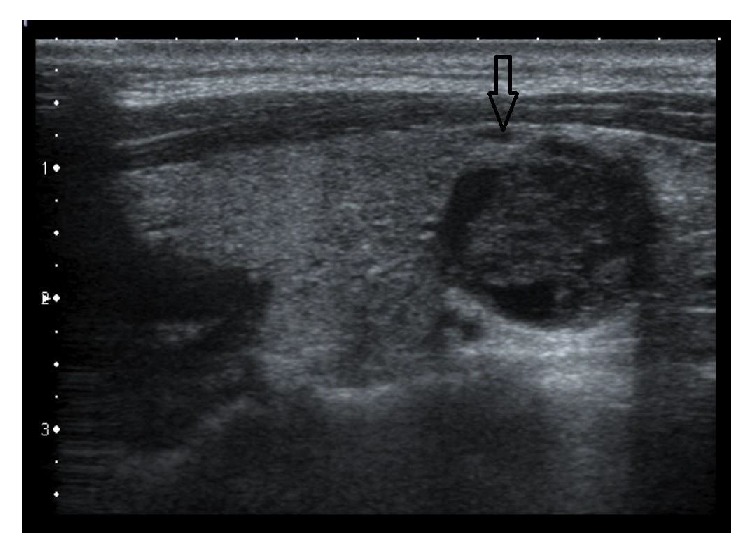
Ultrasound neck showing a well-defined hypoechoic parathyroid adenoma (arrow).

Blood investigation showed a normal serum creatinine level of 60 *μ*mol/L, elevated calcium level of 3.16 mmol/L (*N* = 2.20–2.50), and moderately decreased phosphorus level of 0.85 mmol/L (*N* = 0.97–1.81). Parathyroid hormone (PTH) was checked and revealed elevated level of 305 ng/L (*N* = 8–49). Moreover, the patient had a neck ultrasound showing multiple parathyroid adenomas (Figure 2) responsible for hyperparathyroidism leading to hypercalcemia and secondary bone lesions. The urine calcium level was also elevated with calcium/creatinine ratio of 1.6 mmol/mmol (*N* = 0.16–0.50). Genetic counseling found a mutation in CDC73 (HRPT2).

The patient had a parathyroidectomy that led to normalization of the calcium level within 72 hours (2.6 mmol/L). PTH level decreased to its normal value (45 ng/L) after one week of the surgery. His renal colic attacks became less frequent. Follow-up renal ultrasound three months later noted a decrease in the number of kidney stones and complete spontaneous disappearance of stones at the end of the first year.

MAG-3 scintigraphy revealed a rapid contrast evacuation with normal kidney function. Patient was not operated on for the UPJO and renal ultrasound on one-year follow-up showed normal findings.

## 3. Discussion 

Congenital ureteropelvic junction obstruction is the most common cause of upper urinary tract obstruction in children. By definition, the diagnosis of UPJO signifies functionally impaired transport of urine from the renal pelvis into the ureter. Because the increased renal pelvic pressure from obstruction may lead to progressive renal injury and impairment, correct diagnosis is clinically important. The impairment may be primary or secondary in nature. This, along with the chronicity and severity of the condition, dictates the course of management [7, 8].

Routine antenatal ultrasonography readily recognizes the presence of hydronephrosis and this has led to earlier detection of UPJO [9]. Although the majority of cases are discovered in the neonatal period, many are still diagnosed later in life manifested with hematuria, kidney stones, or abdominal discomfort. The outcome usually is either improvement in the dilatation after birth or worsening with time which eventually may require surgical intervention [10].

Nuclear medicine scanning may be used to quantitatively assess the differential renal function. It has become a primary study for defining ureteropelvic junction obstruction (UPJO) and establishing the decision for surgery [11]. In our case, MAG3 noted bilateral rapid evacuation without abnormal findings giving us some advantage to avoid surgery for the right UPJO and safely monitor the period after parathyroidectomy until the complete dissolution of all kidney stones.

Parathyroid adenoma, which is responsible for primary hyperparathyroidism (PHPT) rarely, occurs in children with an estimated incidence of 2–5 cases per 100,000 of population. PHPT is most often sporadic but may also be seen with hyperplasia of the glands in patients with multiple endocrine neoplasia (MEN) I. Most of the patients with PHPT present with bone diseases mainly fractures or rickets [12, 13]. Although primary hyperparathyroidism is rare in children, presence of urolithiasis or any symptoms suggestive of urolithiasis such as hematuria in this age group should trigger the investigation and exclusion of parathyroid abnormalities [14], taking into account the fact that 50% of patients who are younger than 30 years and diagnosed with primary hyperparathyroidism have urolithiasis [15]. In addition, another study has concluded that 6% of children with urolithiasis had primary hyperparathyroidism [16].

In a view of UPJO and concurrent renal calculi in pediatrics patients, a long-term follow-up study has found that an identifiable metabolic etiology was found in the majority of cases and it suggested that the presence of metabolic abnormality significantly predisposes to recurrent nonstruvite renal lithiasis in such cases [17]. Patients with congenital UPJO and associated nephrolithiasis are found to have higher rate of metabolic abnormalities compared to those with nephrolithiasis not associated with UPJO. This finding supports the fact that urinary stasis alone cannot explain stone formation in patients with UPJO, prompting the need for further metabolic screening and investigations in cases of UPJO and nephrolithiasis [18]. The role of metabolic risk factors in the formation of renal calculi should be investigated, even in the picture of congenital UPJO; and the point of abnormal urinary biochemistry adding a role in the high incidence of nephrolithiasis in children with urinary tract anomalies should prompt a screening of urinary and serum biochemistry in these patients [19].

The incidence of renal calculi in patients with ureteropelvic junction obstruction (UPJO) is nearly 20% [17]. Soylu et al. suggest that it may be due to pelvic dilatation and urine stasis [20]. Our patient remained asymptomatic with his UPJO for a period of fourteen years, before manifesting with a right flank pain due to kidney stones that aggravated the underlying pelvic dilation. The presence of nephrolithiasis directed us towards metabolic work-up that later revealed hypercalcemia which was caused by a parathyroid adenoma leading to PHPT.

A linkage between hyperparathyroidism-jaw tumor syndrome (HPT-JT), which is linked to a chromosomal mutation in HRPT2, and renal diseases was published in a previous study, where two families with HPT-JT syndrome were followed and found to have adult renal hamartomas and cystic kidney disease as prominent features; and this possibly represented a new phenotypic variant of the HPT-JT syndrome. In one of the families, renal lesions were even more prominent, as five out of six individuals had renal lesions, while hyperparathyroidism was found in four individuals and jaw tumor was found in two individuals only [21]. What is interesting about our case is that symptomatic kidney stones led to the diagnosis of PHPT which was secondary to a mutation in CDC73 (HRPT2). PHPT can potentially be the only or the main cause of kidney stones since our patient had bilateral kidney stones despite having only unilateral right dilated pelvis. However, it is possible that UPJO is a second triggering factor that accelerated the formation of kidney stones and other related symptoms.

We believe that association of UPJO and PHPT is a rare coincidence that can be linked as a congenital anomaly and genetic mutation, role to be more investigated. Careful work-up of children with ureteropelvic junction obstruction who develop unilateral or bilateral urolithiasis is recommended to exclude a concomitant metabolic disease. Presence of kidney stones in UPJO is not always an indication for surgery mainly in the absence of renal function impairment. Treatment of the primary cause and close monitoring can lead to complete dissolution of kidney stones.
